# Coupled Human-Environment Dynamics of Forest Pest Spread and Control in a Multi-Patch, Stochastic Setting

**DOI:** 10.1371/journal.pone.0139353

**Published:** 2015-10-02

**Authors:** Qasim Ali, Chris T. Bauch, Madhur Anand

**Affiliations:** 1 Department of Applied Mathematics, Western University, London, Ontario, Canada; 2 Department of Mathematics and Statistics, University of Guelph, Guelph, Ontario, Canada; 3 Department of Applied Mathematics, University of Waterloo, Waterloo, Ontario, Canada; 4 School of Environmental Sciences, University of Guelph, Guelph, Ontario, Canada; Natural Resources Canada, CANADA

## Abstract

**Background:**

The transportation of camp firewood infested by non-native forest pests such as Asian long-horned beetle (ALB) and emerald ash borer (EAB) has severe impacts on North American forests. Once invasive forest pests are established, it can be difficult to eradicate them. Hence, preventing the long-distance transport of firewood by individuals is crucial.

**Methods:**

Here we develop a stochastic simulation model that captures the interaction between forest pest infestations and human decisions regarding firewood transportation. The population of trees is distributed across 10 patches (parks) comprising a “low volume” partition of 5 patches that experience a low volume of park visitors, and a “high volume” partition of 5 patches experiencing a high visitor volume. The infestation spreads within a patch—and also between patches—according to the probability of between-patch firewood transportation. Individuals decide to transport firewood or buy it locally based on the costs of locally purchased versus transported firewood, social norms, social learning, and level of concern for observed infestations.

**Results:**

We find that the average time until a patch becomes infested depends nonlinearly on many model parameters. In particular, modest increases in the tree removal rate, modest increases in public concern for infestation, and modest decreases in the cost of locally purchased firewood, relative to baseline (current) values, cause very large increases in the average time until a patch becomes infested due to firewood transport from other patches, thereby better preventing long-distance spread. Patches that experience lower visitor volumes benefit more from firewood movement restrictions than patches that experience higher visitor volumes. Also, cross–patch infestations not only seed new infestations, they can also worsen existing infestations to a surprising extent: long-term infestations are more intense in the high volume patches than the low volume patches, even when infestation is already endemic everywhere.

**Conclusions:**

The success of efforts to prevent long-distance spread of forest pests may depend sensitively on the interaction between outbreak dynamics and human social processes, with similar levels of effort producing very different outcomes depending on where the coupled human and natural system exists in parameter space. Further development of such modeling approaches through better empirical validation should yield more precise recommendations for ways to optimally prevent the long-distance spread of invasive forest pests.

## Introduction

Infestation of North American forests by non–native insect species like Emerald ash borers (EAB), Asian long–horn beetles (ALB) and Citrus long–horn beetles (CLB) has had severe impacts on many ecologically and economically important tree species, such as Ash trees [1]. These non–native forest pests were often first introduced through international trade in the 1990s and 2000s or before [1,2]. Since their introduction, these forest pest species have subsequently spread widely in North American regions, causing the death of billions of trees, and thereby extensive economic and environmental damage [1,3]. An estimated $17.6 million were spent by the Canadian government between 2005 and 2010 in order to treat, remove and replace the infested trees, and it is thought that this amount could reach $2 billion dollars in coming decades [4]. This situation is much worse in several parts of the United States, particularly in the Midwestern states, where the projected cost of eradicating infestations and planting new ash trees is estimated at $26 billion [1,5]. Invasive forest pests arguably represent one of the most significant threats to the region’s natural forests and domestic parks [3]. Forest pest infestations represent a clear case of an ecological and economic catastrophe that needs to be rapidly addressed.

In previous empirical studies it has been found that the spread of forest pests occurs either due to natural dispersal, where insects disperse locally under their own power, or due to human–assisted dispersal, where the wood of infested trees is transported from an infested region to a non–infested region [6,7]. The natural dispersal rate depends upon the density of insects in the region. In Michigan, the dispersal rate in low density infestations has been estimated to be as low as 300 m/year [8] while in high density infestations it has been estimated to be 16 km/year [9]. However, the rate of spread due to the transportation of Ash tree products (e.g. firewood) can be much higher than the natural dispersal rate [10]. Much long–distance dispersal is due to individuals transporting infested camp firewood to non–infested areas such as national or provincial parks. Reasons for transporting firewood instead of buying it locally include greater convenience and lower costs.

Several mathematical models have been developed to investigate forest pest invasions and explore possible control strategies to mitigate the resulting ecological and economic losses. These models have focused on the spread rate of non–native insects in North America [2,8,11], their possible future impacts [3,12], and their social and financial impacts [13]. Some of these models explore control strategies such as early detection of infestation and control of insects by means of insecticide treatment, and pre-emptive removal of all Ash trees in a region with replacement by non–Ash trees [13–15]. Such measures can help control local infestations. However, in order to prevent long–distance dispersal causing the start of new infestations in previously untouched regions, it is necessary to prevent transport of infested firewood through measures such as expanding public awareness and implementing firewood transport restrictions. Reducing the price of locally purchased firewood may also reduce the spread [10,16]. Preventing long–distance dispersal of these pests requires better understanding of how human decision–making in the social context interacts with forest pest infestations.

Barlow *et al*. have previously developed a deterministic two–patch model of the impact of human decisions on firewood transportation between two hypothetical patches, as well as the impact of forest pest infestations on human decisions. Models that capture the two–way interplay between human decision–making dynamics and natural dynamics are referred to as coupled human–environment system models [17,18]. The two–patch model of Barlow *et al*. included natural processes such as within-patch spread of forest pests and patch–to–patch spread (known as cross–patch infestation spread), and human processes such as social learning, social norms, and economic considerations such as cost of firewood.

Here, we build on the model of Barlow *et al*. to create a stochastic 10–patch model that captures the social, natural and economic aspects of infestation dispersal via firewood transportation. The long-distance dispersal of forest pests is inherently highly variable since the start of an infestation in a given patch can be caused by the arrival of a single bundle of infested firewood, and the probability that the infestation becomes established and spreads depends on a large number of independent factors such as viability of the founder population, environmental conditions, human decisions and other biotic and abiotic factors. In order to approximate this variability, we use a stochastic model. This enables us to produce new outcome variables such as time–to–first–cross–patch–infestation (the time that elapses before infestation spreads from one patch to another patch). We determine how infestation control depends on human and natural parameter values, using a multi–patch park network topology that qualitatively resembles that of the Ontario Park system. Our objective was to develop a qualitative understanding of the range of dynamics exhibited by such systems and to illustrate how these systems can exhibit nonlinear feedbacks. Therefore, the model was formulated for simplicity and generality, and to capture the qualitative features of forest pest infestations and firewood movement patterns as observed in various recent forest pest outbreaks, rather than representing a particular species in a particular geographic location.

## Methods

### 2.1 Geography of Ontario Parks

The interactions between forest pest invasions and human decisions regarding firewood transportation can be better understood in the context of the spatial distribution of Ontario Parks (the provincial park system of Ontario, Canada). The spatial distribution of parks and the strength of connections between parks can have a large impact on cross–park (“cross–patch”) infestation spread.

Ontario Parks can be divided into two categories: operating parks that are regulated under the provincial authority, and non–operating parks that have no fees/staff and only limited facilities. According to Ontario Parks statistics from 2010, the southern and central regions of Ontario are the most popular places for day–use visitors and overnight campers [19]. These regions have 75% to 80% of the total visitors of all Ontario parks per year [19]. Large populations visit the central region of Ontario, and these may include visitors from northern and southern regions. In research on the attitudes of visitors regarding use of left-over (residual) firewood at Wisconsin State parks, it was found that visitors take up to 15% of unused firewood back to their homes [20]. If this firewood is infested it could become the seed of a new local infestation.

Ontario Parks are numerous and highly connected to one another, presenting a complicated geometrical structure [21]. With respect to the movement of camp firewood, the parks are connected through visits from the public. The high connectivity between parks stems from the fact that any given park may receive visitors who reside anywhere in Ontario, any of whom may bring along firewood that was purchased at their point of origin.

**Fig 1:**
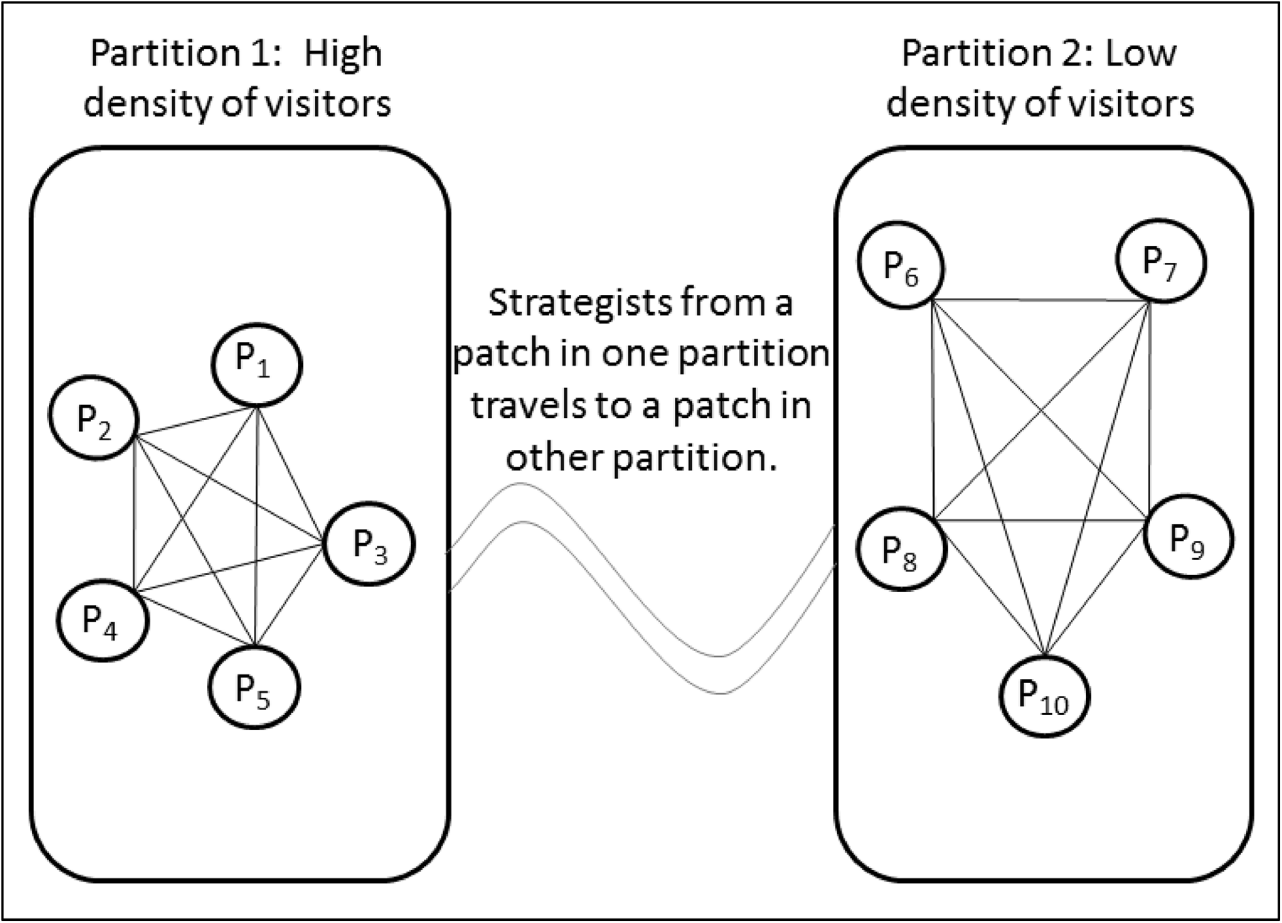
Patch geometry used for the simulation model. Partition 1 patches experiences a high visitor volume while Partition 2 patches experiences a low visitor volume. Transport strategists move firewood between patches in both partitions.

Such high connectivity can be approximated by a mesh topology (Fig 1). The parks in the central region of Ontario are most dense and experience the highest volume of visitors. The southeastern region has a high density of parks as well but the visitor volume is less than the visitor volume of central parks. The parks in these regions are located mostly inland. In contrast, the southwestern parks distributed linearly along lakesides have a slightly higher volume of visitors than southeastern parks. Northern parks exhibit the same tendency for inland locations however the density of parks and the volume of visitors are significantly lower than the parks in central and southern regions.

Here, we simplify this structure to capture the basic distinction between centrally located parks with high visitor volumes, and proximal parks with low visitor volumes. We divide Ontario parks into two partitions: one for the southern and central Ontario, which receives a high volume of visitors, and the other for Northern Ontario, which receives a low volume of visitors. Each patch in either partition is connected with all patches in that partition as well as with all patches in the other partition as shown in Fig 1. Hence, the total system is comprised of completely connected patches while patches are placed in either of the partitions according to their volume of visitors. Moreover, the strength of connection varies depending on whether patches are in the high volume or low volume partition: patches in the low volume partition receives a lower volume of visits from other patches in the same partition as well as a lower volume of visits from patches in the high volume partition.

### 2.2 Model

We developed a discrete–time, mechanistic, stochastic model to study pest invasions, human decision–making, and firewood movement in the 10–patch system. The simulation time step is one week. Each patch has a population of *S*_i_(t) susceptible trees and *I*_i_(t) infested trees. The total carrying capacity of susceptible and infested trees is *K*. Susceptible trees become infested trees according to specific transition probabilities based on assumed transmission mechanisms (details are below). Susceptible trees become infested with a given probability per time step due to local infestation (infestation within a patch) according to “standard incidence” transmission mechanism. Susceptible trees also become infested due to firewood transport from other infested patches, depending on the prevalence of infestation in the other patch, visitor volumes between the patches, and human decisions regarding whether or not to transport firewood between the patches. Infested trees die with a certain probability per time step, and in the model notation use *‘R’* to denote a ‘removed’ state, for trees that have died after being infested.

Each patch is considered to include both a local park as well as the local residents of that area. Hence, the individuals who live in patch *i* can either be local strategists (where *L*_i_(t) represents the proportion of local strategists in patch *i*) who always buy and burn firewood at the patches they visit, or they can be transport strategists (where *T*_i_(t) represents the proportion of transport strategists in patch *i*) who always bring firewood from their home patch *i* when they visit one of the other 9 patches (and thus risk spreading the infestation, if patch *i* is infested). Thus, we can write the relation between the proportion of local and transport strategists as *L*_i_(t) = 1 –*T*_i_(t).

In particular, the probability per time step that a susceptible tree becomes infested due to local (within–patch) dispersal is

$$P_{\text{S to I,i}}\;(t)=\beta \frac{I_{i}(t)}{K}$$ (1)

where *β* is the transmission probability constant, *I*_*i*_(t) is the number of infested trees in patch *i*, and *K* is the carrying capacity in patch *i* (Table 1). This form of the transmission probability, where the infestation rate depends on the density of infested trees, is referred to as standard incidence [22]. We note that the time dependence of *P*_S to I,i_ occurs due to its dependence on the variable *I*_i_(*t*), representing the number of infested trees at time *t*. The nonlinearity of the simulation model stems from Eq 1, since the probability of infestation *P*_StoI,i_ depends on the infestation prevalence *I*_*i*_(t)/*K* instead of being a constant value, therefore the mean of the binomial distribution for the number of trees that get infected depends on the nonlinear term *S*_*i*_(t)*I*_*i*_(t)/*K*.

**Table 1:** Model variables and transition probabilities.

*S*_*i*_(*t*)	Number of susceptible trees at time *t* in *i* ^th^ patch.
*I*_*i*_(*t*)	Number of infested trees at time *t* in *i* ^th^ patch.
*U*_*L*_	Utility for local strategists.
*U*_*T*_	Utility for transport strategists.
*L*_i_(t)	Proportion of local strategists at time *t* in *i* ^th^ patch.
*T*_i_(t)	Proportion of transport strategists at time *t* in *i* ^th^ patch.
P_S to I_	Probability that a susceptible tree becomes infested due to within-patch infestation.
P_0 to S_	Probability that a susceptible tree is recruited.
P_I to R_	Probability that an infested tree dies in a given time step.
P_L to T_	Probability that a local strategist becomes a transport strategist.
P_T to L_	Probability that a transport strategist becomes a local strategist.
P_S to IT_	Probability that a susceptible tree gets infested due to cross–patch firewood transport.
*num*_I to R_	Number of infested trees that die in a given time step.
*num*_S to I_	Number of susceptible trees that get infested due to within-patch infestation in a given time step.
*num*_0 to S_	Number of susceptible trees that are recruited in a given time step.
*num*_L to T_	Number of local strategists that become transport strategists in a given time step.
*num*_T to L_	Number of transport strategists that become local strategists in a given time step.
*num*_S to IT_	Number of susceptible trees that get infested due to cross-patch infestation in a given time step.

The total number of susceptible trees that become infested per time step, *num*_StoI_(t), can be found from the probability *P*_StoI_(t) by sampling from a binomial distribution according to

$$num_{\text{S to I}}(t)=Binomial(S_{i}(t),P_{\text{S to I}}(t)),$$ (2)

Once a susceptible tree becomes infested, it survives for an average time duration *D*. Thus, an infested tree has a probability

$$P_{\text{I to R}}=\varepsilon =\frac{1}{D}$$ (3)

per time step of dying. Thus, the number of trees *num*_I to R_ that die in each time step can be written as

$$num_{\text{I to R}}(t)=Binomial(I_{i}(t),P_{\text{I to R}}),$$ (4)

New trees can grow in the place of dead trees. However, their growth is subject to the availability of space and the growth rate (fecundity) *r* of susceptible trees. Therefore, if we assume that (*I*_i_(t) + *S*_i_(t)) / *K* is the proportion of trees already existing in the patch, then 1 –(*I*_i_(t) + *S*_i_(t)) / *K* would be the available space for new trees to grow. Therefore, the probability *P*_0 to S_ that a susceptible tree gives rise to a (susceptible) offspring is

$$P_{0\;\text{to S}}\;\left(t\right)=r\left(1-\frac{I_{i}\left(t\right)+S_{i}\left(t\right)}{K}\right)$$ (5)

Hence, the number of new trees per time step is

$$num_{0\;\text{to S}}\;\left(t\right)=Binomial(S_{i}\left(t\right),P_{0\;\text{to S}}\;\left(t\right)),$$ (6)

Firewood cost, the influence of pest outbreaks upon decision–making, social norms and imitation dynamics (e.g. social learning) inform decision–making. If the local firewood cost in a patch is higher than the cost of bringing firewood then the individuals visiting from other patches may bring their own firewood instead. However, individuals are also influenced by awareness of the impact of pest outbreaks in their own patch, and emerging rules about social conduct [10]. These social, financial and behavioral factors are together used to define the utility (a quantitative measure of preference) for these strategists, representing the utility for being a local strategist who always “burns where they buy” versus the utility for being a transport strategist who transports firewood before burning it.

In particular, we define the utility for a local strategist as *U*_*L*_(t) while the utility for a transport strategist as *U*_*T*_(t). The equations for *U*_*L*_(t) and *U*_*T*_(t) are

$$\begin{array}{l} U_{L}\left(t\right)=-C_{L}+n(L_{i}\left(t\right)-0.5)\\ U_{T}\left(t\right)=-C_{T}+n(0.5-L_{i}\left(t\right))-fI_{i}\left(t\right) \end{array}\}$$ (7)

where *C*_L_ and *C*_T_ are the local and transport firewood costs respectively and *n* controls the strength of social norms. The parameter *n* can be interpreted as the strength of social pressure in favor of the dominant behavior or attitude [23,24]. If *n* is large and there are many local strategists (*L*_i_(*t*) is high), then *U*_L_(*t*) is high since there is social pressure for individuals to conform to the norm of not transporting firewood, and this tends to further increase *L*_i_(*t*). Conversely, if *L*_i_(*t*) is low, causing *U*_T_(*t*) to be high, then social pressure will further reduce *L*_i_(*t*). The infestation concern parameter *f* is a proportionality constant that controls the extent to which infestation prevalence influences individual decision-making.

The total number of individuals in a patch is a constant *N*_*S*_ = *N*_*L*_(t) + *N*_*T*_(t), where *N*_*L*_(t) is the number of local strategists and *N*_*T*_(t) is the number of transport strategists. Hence, the total number of individuals remains the same throughout the simulation. Accordingly,

$$L_{i}\left(t\right)=\frac{N_{L,i}\left(t\right)}{N_{S,i}}\;\text{and}\;T_{i}\left(t\right)=\frac{N_{T,i}\left(t\right)}{N_{S,i}}$$ (8)

We assume that an individual “samples” (i.e. speaks to) other persons in their own patch regarding firewood transport, firewood cost, and forest pest infestations with a specific probability per unit time [10,25]. Sampling may occur through person-to-person contact or through other means such as social media or telephone. During this interaction, individuals compare their utilities received for their respective strategies. If a sampled person is playing a different strategy and is receiving a higher utility, then the individual doing the sampling switches to the sampled person’s strategy with a probability proportional to the expected gain in utility. Therefore, the total probability per time step that a local strategist becomes a transport strategist is the product of the probability of sampling (the “social learning rate”) and the probability of switching strategies:

$$P_{\text{L to T}}\left(t\right)=\{\begin{array}{cc} 0 & U_{L}\left(t\right)\geq U_{T}\left(t\right)\\ \sigma (U_{T}\left(t\right)-U_{L}\left(t\right)) & U_{L}\left(t\right)<U_{T}\left(t\right) \end{array}$$ (9)

where *σ* represents the social learning rate multiplied by a proportionality constant that guarantees *P*_L to T_ < 1. Since we only vary the social learning rate in this analysis, we will treat changes in *σ* as equivalent to changes in the social learning rate, for all practical purposes. Similarly, the rate at which a transport strategist becomes a local strategist through sampling is

$$P_{\text{T to L}}\;\left(t\right)=\{\begin{array}{cc} 0 & U_{L}\left(t\right)\leq U_{T}\left(t\right)\\ \sigma (U_{L}\left(t\right)-U_{T}\left(t\right)) & U_{L}\left(t\right)>U_{T}\left(t\right) \end{array}$$ (10)

The number of individual changing strategies in each time step is then

$$num_{\text{L to T}}\;\left(t\right)=Binomial(N_{L,i}\;\left(t\right),P_{\text{L to T}}\;\left(t\right)),$$ (11)

$$num_{\text{T to L}}\;\left(t\right)=Binomial(N_{T,i}\left(t\right),P_{\text{T to L}}\;\left(t\right)),$$ (12)

Just as infestation influences human behavior, human behavior regarding firewood transportation influences infestation spread between patches. The probability that a susceptible tree in a given patch gets infested due to transported firewood depends upon the proportion of transport strategists in neighboring infested patches and the amount of travel (and thus firewood movement) between patches. Thus the probability of cross–patch infestation occurring in patch *i* due to infested firewood from patch *j* can be written as,

$$P_{\text{S to IT},j}\;\left(t\right)=\beta T_{i}\left(t\right)\frac{I_{i}\left(t\right)}{K}\{\begin{array}{cc} \;d_{H} & i=1..5\;(\text{within P}1)\\ \;d_{L} & \text{Otherwise}\;(\text{within P}2\;\text{and between P}1\;\text{and P}2) \end{array}\;,\;i\neq j$$ (13)

where *d*_H_ > *d*_L_ since the volume of visitors within Partition 1 (P1) is significantly higher than either the volume of visitors within Partition 2 (P2) or the volume of visits between the two partitions.

Based on these transmission probabilities, the number of new infestations in patch *i* per time step due to cross–patch movement of firewood is the sum of all cross–patch infestations introduced from patches *j* ≠ *i*:

$$num_{\text{S to IT,i}}\;(t)=\sum_{j\neq i}Binomial(S_{i}(t),P_{\text{S to IT,j}}\;\left(t\right)),$$ (14)

Cross–patch infestation can occur several times during the simulation. However, the first cross–patch infestation event is most important since it forms the nucleus of the first outbreak in a previously un-infested patch. Thus, an important outcome variable is time–to–first–cross–patch–infestation, defined as the time between the start of the simulation and the time that a patch first becomes infested due to firewood transport from another patch.

Once the number of state transitions is computed from the binomial sampler at each time step from Eqs 2, 4, 6, 11, 12 and 14, the state variables *S*_i_(t), *I*_i_(t), *L*_i_(t), and *T*_i_(t) are updated. The number of susceptible trees increases with the growth of new susceptible trees and decreases due to infestations originating either inside the patch or from other patches, thus:

$$S_{i}(t+1)=S_{i}(t)-num_{\text{S to I,i}}\;\left(t\right)+num_{0\;\text{to S,i}}\;\left(t\right)-num_{\text{S to IT,i}}\;\left(t\right)$$ (15)

The infested trees increase correspondingly increase due to spread of infestation, but their number decreases when infested trees die:

$$I_{i}(t+1)=I_{i}(t)+num_{\text{S to I}}\;\left(t\right)-num_{\text{I to R}}\;\left(t\right)+num_{\text{S to IT}}\;\left(t\right)$$ (16)

Finally, transitions between the numbers of local and transport strategists are given by

$$N_{L,i}(t+1)=N_{L,i}(t)-num_{\text{L to T}}\;\left(t\right)+num_{\text{T to L}}\;\left(t\right)$$ (17)

$$N_{T,i}(t+1)=N_{T,i}(t)+num_{\text{L to T}}\;\left(t\right)-num_{\text{T to L}}\;\left(t\right)$$ (18)

### 2.3 Parameterization

Baseline parameter values were obtained from empirical data concerning tree species, forest pest infestations, and firewood costs (Table 2). Many parameter values were borrowed from the previous 2-patch deterministic model [10]. The ecological parameters, i.e. fecundity of trees *r*, differs for various trees species infested by EAB and ALB, while the probability of transmission *β* and mortality probability per time step of infested trees *ε* depends upon the density of insects in the patch. These parameters are well studied in the literature [7,10,11,19,26–28]. We used the empirical results found in [26] to estimate the fecundity of trees *r* = 0.06 / year by multiplying the mean survival of trees species, i.e. 2.4 ·10^−6^ trees / seed, with the total number of seeds · tree^-1^ · year^-1^, i.e. 25,000. The transmission probability parameter *β* was determined by a simple spread model to approximate the insect’s arrival time in a patch. Various spread rates have been estimated in the literature, ranging from low, i.e. 10 km/year, to high, i.e. 50 km/year, [28,29]. The parameter was determined by adjusting the distance covered by insects according the area covered. We assumed 5 ha = 50000 *m* ^2^ of land with a spread rate of 25000 *m* ^2^ / year, yielding the transmission probability parameter *β* = 0.5 / year. The fatality probability *ε* = 1/3 per year was taken from literature [27] stating that it takes three years, on average, for a tree infested by EAB to die in recent infestations in Michigan and Ontario. The economic parameters for the cost of local and transported firewood, *C*_L_ and *C*_T_ respectively, vary by region. However, in the case of Ontario, baseline values for the parameters *C*_L_ = $ 6.75 and *C*_T_ = $ 5.00 were determined by surveying park administration offices and surveying the local markets [10].

**Table 2:** Parameter definitions and their baseline values.

Symbols	Definitions	Parameter values	References
*R*	Fecundity of trees per unit time	0.06year	[26]
*β*	Transmission probability per unit time	5×10-1year	[28,29]
*d*_H_	Proportionality constant controlling volume of visitors to a given patch in high volume partition P1	0.02	[19]
*d*_L_	Proportionality constant controlling volume of visitors to a given patch in low volume partition P2, or volume between P1 and P2	0.003	[19]
*ε*	Probability of death of infested tree per unit time	13year	[27]
*C*_L_	Cost of buying bundle of local firewood	$ 6.75	[10]
*C*_T_	Cost of buying bundle of transported firewood	$ 5.00	[10]
*N*	Strength of social norms	0.1	–
*F*	Population sensitivity to infestations (impact of outbreaks upon decisions)	0.1	–
*K*	Carrying capacity in a patch	5000	[10]
*σ*	Social learning probability per unit time	0.1year	–
Δ*t*	Time step	152year	[10]
*N*_*S*_	Total number of strategists per patch	1000	–

System–specific empirical data were not available for some sociological parameters (e.g. *n*, *f*, *σ*) hence baseline values for these parameters were calibrated until ecologically and sociologically plausible dynamics were obtained (Table 2). In particular, we sought model dynamics consistent with recent outbreaks with EAB and ALB in Ontario and other jurisdictions: infestation generates some level of concern in the population, but the concern is not sufficient to prevent regional spread, which occurs on the timescale of several years. Moreover, infestation spreads more quickly to patches that experience a high volume of visitors than patches that experience a low volume of visitors. Univariate sensitivity analysis was used to study the impact of varying social influence *n*, social learning *σ*, outbreaks *f*, fatality rate *ε*, firewood cost *C*_L_ and controlling rate *d*, by changing these parameters one at a time while the other parameters remained at their baseline values.

## Results

For each set of parameter values, 100 stochastic realizations were generated and the mean and two standard deviations thereof were computed. The model was coded in Matlab R2014a using the built–in *binornd* function to generate random variates sampled from a binomial distribution. The time-to-first-cross–patch-infestation, and time series of infected trees and proportions of local and transport strategists, were generated.

### 3.1 Baseline analysis

At the initial time (*t =* 0), only Patch 1 was infested with 15 infested trees, *I*_1_(*t* = 0) = 15, and the remaining trees were susceptible, *S*_1_(*t* = 0) = 4985, while all the other patches were initially not infested (i.e. *I*_*i*_(*t* = 0) = 0 and *S*_*i*_(*t* = 0) = 5000, where *i* = 1..10). Moreover, the population of total strategists N_S_ was fixed at 1,000 in each patch and the proportion of local strategists *L*_*i*_ and transport strategists *T*_*i*_ was initialized as 0.1 and 0.9 respectively, (i.e. *N*_*L*,*i*_ (0) = 100 and *N*_*T*,*i*_ (0) = 900).

**Fig 2:**
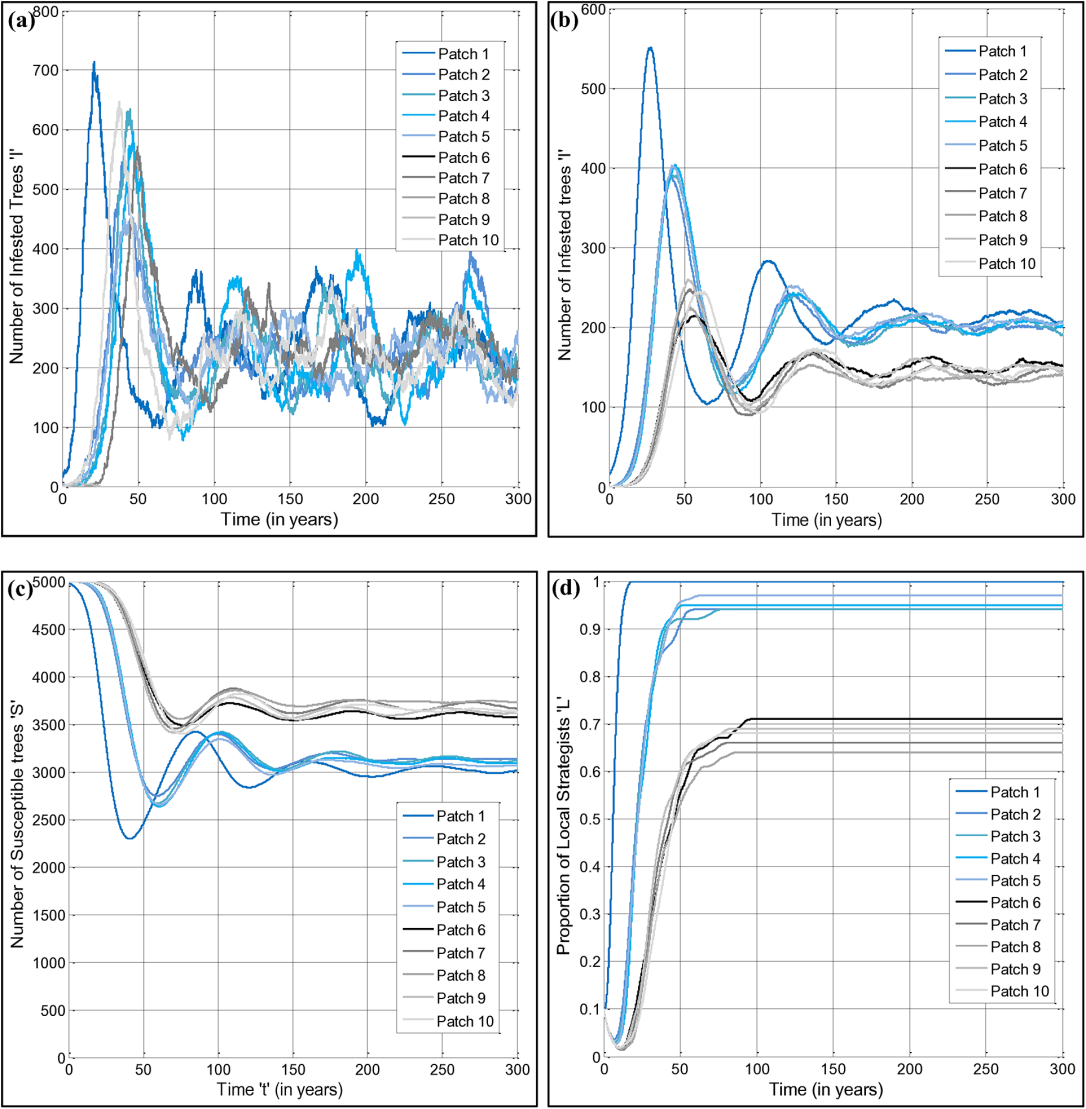
Dynamics of infested trees, susceptible trees, and local strategists. Panels show a single realization of the number of infested trees (a); the average of 100 realizations of the number of infested trees (b), the number of susceptible trees (c), and the proportion of local strategists (d), over 300 years of simulated time. Only patch 1 was infested at time *t* = 0. Patches from 1 to 5 are in the low visitor volume Partition 1 while patches from 6 to 10 are in the high visitor volume Partition 2. The parametric values are given in Table 2.

The stochastic nature of the model dynamics is observed by simulating the number of infested trees against time in a single realization (Fig 2A, data available in the supplementary material: S1 Matlab Data File). Patch 1, where the infestation starts, experiences an outbreak that peaks rapidly. As the number of infested trees in patch 1 grows, the probability of infected firewood being transported from patch 1 to other patches increases. Eventually, after a few years, the other patches start to experience outbreaks as a few bundles of infested firewood make their way to those patches and a viable local population of forest pests is formed. In general, patches 2–5 (the high volume partition P1) experience outbreaks sooner than patches 6–10 (the low volume partition P2), except that patch 10 is the next patch to experience an outbreak, after patch 1, for this particular realization (Fig 2A). In contrast, patch 9 avoids infestation throughout the entire simulation. This illustrates the stochastic nature of long–distance forest pest spread. For all other patches, the infestation settles down to a steady state where the destruction of susceptible trees through infestation is balanced by the creation of susceptible trees through recruitment, in the long-term. We note that we ignore the impact of local control measures, since our focus is on long–distance establishment of new infestation sites. However, local control could be represented by increasing the parameter *ε* representing the probability of death of infested trees per unit time, and we investigate this change in the sensitivity analysis.

The expected features of multi–patch transmission are clear in time series of the average number of infested trees in each patch over 100 realizations (Fig 2B). We observe that, on average, patch 1 experiences an outbreak with a higher peak, on account of the higher initial number of infested trees. On average, patches 2–5 in the high volume partition experience outbreaks next, and patches 6–10 in the low volume partition experience outbreaks last. The equilibrium infestation levels are also higher in the high volume partition, due to more frequent instances of cross–patch infestation. Hence, the patches in the low volume partition benefit not only from delayed introduction of new infestations, but also the fact that subsequent re–introductions of the infestation are less, which reduces the equilibrium number of infested trees in those patches as well, to a surprising extent. The plot of the average number of susceptible trees in each patch over time (Fig 2C) shows activity that mirrors the plots of the average number of infected trees in each patch over time (Fig 2B).

While outbreaks are unfolding in the 10 patches, there are also changes to the relative proportions of local and transport strategists in the patches. This strongly influences the cross–patch infestation dynamics (Fig 2D). The outbreak in patch 1 is severe (Fig 2B), and this results in a relatively quick response in the human population, with a rapid increase in the proportion of local strategists to 100% over a 20–year period (Fig 2D). Initially, the proportion of local strategists increases due to the *f·I* term, as a result of the human population’s response to the infestation that causes transport strategists to become local strategists. Subsequently, over a decade, as the behavior of purchasing local firewood becomes widespread and awareness of infestation spreads, the local strategy establishes itself as a new social norm and becomes self–perpetuating, pushing the proportion of local strategists to even higher equilibrium levels. However, in present-day real populations, awareness of forest pest infestations is more limited and social norms remain relatively weak. This situation is analogous to the transitions regarding norms that govern second–hand smoke over the past few decades. Many populations shifted from a view that second–hand smoke is tolerable, to a view that exposing others to second–hand smoke is a harmful behavior; this illustrates how new social norms can develop that would have been difficult to conceive a few decades ago [30,31].

The outbreaks in patches 2–5 are somewhat less severe, and thus the proportion of local strategists increases more slowly and to a lower equilibrium level (Fig 2D). Finally, the outbreaks in patches 6–10 are delayed the most, and are least severe, and thus the number of local strategists grows slowest, and reaches the lowest equilibrium level. In the long–term, local strategists are widespread in all 10 patches and help reduce additional cross–patch infestation events, especially in the high volume patches. On average, human populations do not react quickly enough to prevent spread to all 10 patches. This baseline scenario reflects recent experience with many forest pest infestations in North America, which have become widespread [1,3]. However, in 4% of model realizations over a simulated time horizon of 50 years in the low density partition, one patch manages to infestation on account of local strategists increasing quickly enough in the other patches to prevent it being infested.

**Fig 3:**
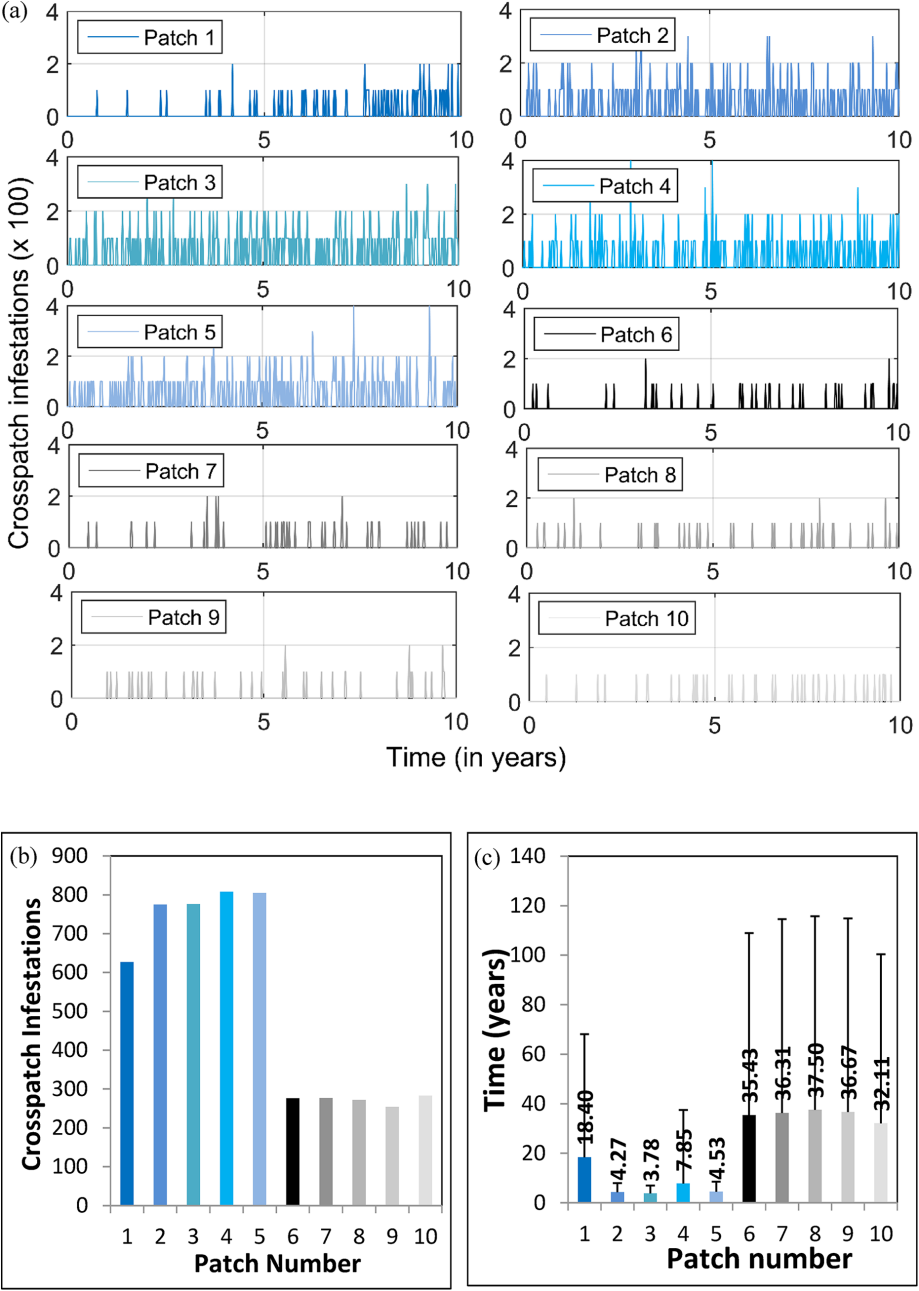
Statistics on number and timing of cross-patch infestation events. The results are averaged across 100 realizations. Patches 1 to 5 are in Partition 1 while patches 6 to 10 are in Partition 2. The parameter values are taken from Table 2. (a) Number of cross–patch infestations experienced by each patch due to the transportation of firewood during the first 10 years of the simulation, in 100s; (b) total number of cross–patch infestations occurring during the 300 years’ simulation time in each patch; (c) mean and two standard deviation (error bars) of the time-to-first-cross–patch-infestation occurring in each patch during the 300 years’ simulation time. Horizontal axis represents the patch number while vertical axis represents the time-to-first-cross–patch-infestation.

The temporal patterns of cross–patch infestation are clarified by plotting the number of cross–patch infestation events experienced by each patch versus time, for all 10 patches in the first 10 years, and including all 100 realizations (Fig 3A, data available in the supplementary material: S1 Matlab Data File), as well as the total number of cross–patch infestations experienced by each patch over 300 years, averaged over all 100 realizations (Fig 3B).

The time delays introduced by the stochastic nature of cross–patch infestation are made clear. Patches 6–10 tend to experience later initial infestations, with few of the 100 realizations yielding an infestation before a few years have passed (Fig 3A). (Patch 1 also experiences later first infestations from other patches, simply because it must infect other patches first, before experiencing a return infestation.) Patches 2–5 experience their first infestations earlier due to their higher volume of visitors from patch 1 residents. The frequency of cross–patch infestations increases with time, as the outbreaks outpace the populations’ social response to the outbreaks (Fig 3A). A similar pattern is observed with respect to the average total number of cross–patch infestations experienced over 300 years (Fig 3B). High volume patches experience between 600 and 800 total cross–patch infestation events on average, whereas low volume patches experience only 250 to 300 cross–patch infestation events (Fig 3B).

Table 3 summarizes the total number of cross–patch infestations over 300 years, stratified by patch of origin of the infestation. The table shows that the number of infestation events is highest for patches in the high volume partition, as expected.

**Table 3:** Number of cross-patch infestation events due to firewood transportation between patches. Patches 1–5 are in Partition 1 while Patches 6–10 are in Partition 2. Columns represent the patches being infested by other patches (e.g. patch (column) 1 is infested by patch (row) 4 on average 1.65 times during the simulation time horizon) and each row represents a patch causing infestations in other patches. Diagonal values are zero by definition. The last column represents the total number of infestation events spread by row-patches while last row represents the total number of infestation events received from column-patches. Data available in the supplementary material: S1 Matlab Data File.

Patch #	1	2	3	4	5	6	7	8	9	10	Total infestations spread
**1**	0	1.94	2.25	1.85	2.04	0.31	0.31	0.31	0.27	0.27	**9.55**
**2**	1.97	0	1.97	2.35	2.2	0.25	0.24	0.45	0.29	0.42	**10.14**
**3**	1.76	2.16	0	2.46	2.51	0.43	0.46	0.31	0.37	0.33	**10.79**
**4**	1.65	2.26	2.29	0	2.17	0.43	0.34	0.37	0.42	0.37	**10.3**
**5**	1.85	2.21	2.23	2.52	0	0.41	0.43	0.32	0.38	0.47	**10.82**
**6**	0.23	0.21	0.19	0.26	0.22	0	0.22	0.31	0.24	0.32	**2.2**
**7**	0.16	0.23	0.14	0.19	0.23	0.34	0	0.33	0.26	0.35	**2.23**
**8**	0.15	0.21	0.23	0.21	0.25	0.21	0.27	0	0.26	0.24	**2.03**
**9**	0.18	0.2	0.19	0.2	0.22	0.23	0.37	0.23	0	0.25	**2.07**
**10**	0.17	0.21	0.2	0.25	0.22	0.32	0.27	0.26	0.23	0	**2.13**
**Total infestations received**	**8.12**	**9.63**	**9.69**	**10.29**	**10.06**	**2.93**	**2.91**	**2.89**	**2.72**	**3.02**	

Finally, the average time-to-first-cross–patch-infestation (*t*_cross_) across the 100 realizations was plotted for each patch (Fig 3C), showing that on average, the high volume patches experience their first infestation event within 4–7 years (except for patch 1, which experiences its first event after 18 years since it must wait for return infestations from other patches). However, on average, the low volume partition patches do not experience their first infestation until 32–38 years.

### 3.2 Sensitivity analysis

In our sensitivity analysis we focus on the time-to-first-cross-patch infestation outcome measure *t*_cross_, since our emphasis is on understanding how to prevent long–distance spread of infestations through firewood movement, rather than on control of local, existing infestations. We explore the impact of changes to the local firewood cost *C*_*L*_, fatality probability per unit time *ε*, infestation concern parameter *f*, visitor volume constants *d*_H_ and *d*_L_, strength of social norms *n* and probability of social learning per unit time, σ. Other parameters are the same as in the remainder of this paper, except that the simulation time horizon is only 100 years instead of 300 years in order to obtain feasible simulation times given our computational constraints.

**Fig 4:**
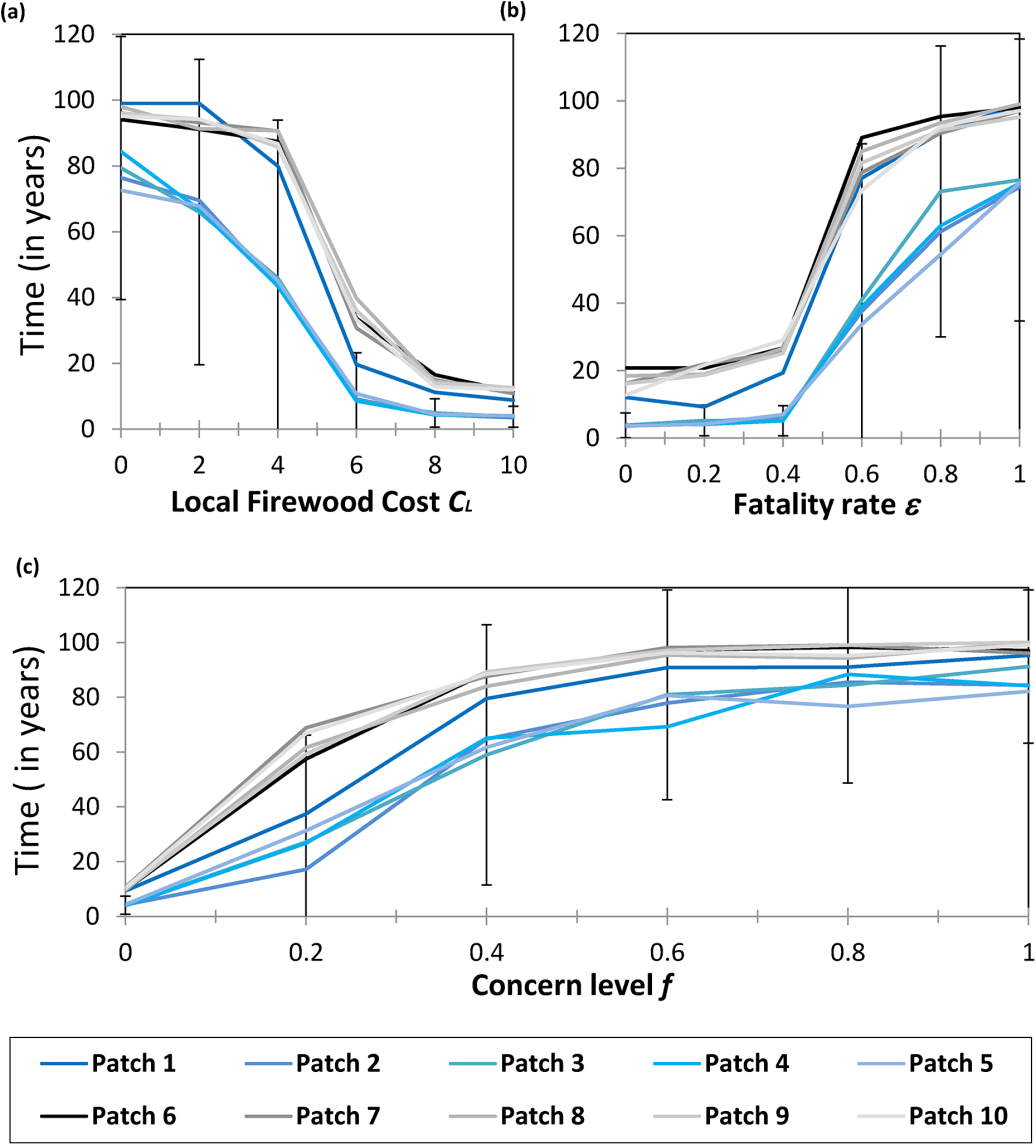
Average of 100 realizations over 100 years of simulated time. Error bars represent two standard deviations from the mean and have been drawn for only Patch 3 to prevent visual clutter (values are similar for other patches). Patches 1–5 are in Partition 1 while patches 6–10 are in Partition 2. See Table 2 for parameter values. Panels show (a) the impact of local firewood cost *C*_*L*_; (b) the impact of fatality probability of trees *ε* upon average *t*_*cross*_; and (c) the impact of the infestation concern parameter *f* upon the average time-to-first-cross–patch-infestation *t*_*cross*_.

**Fig 5:**
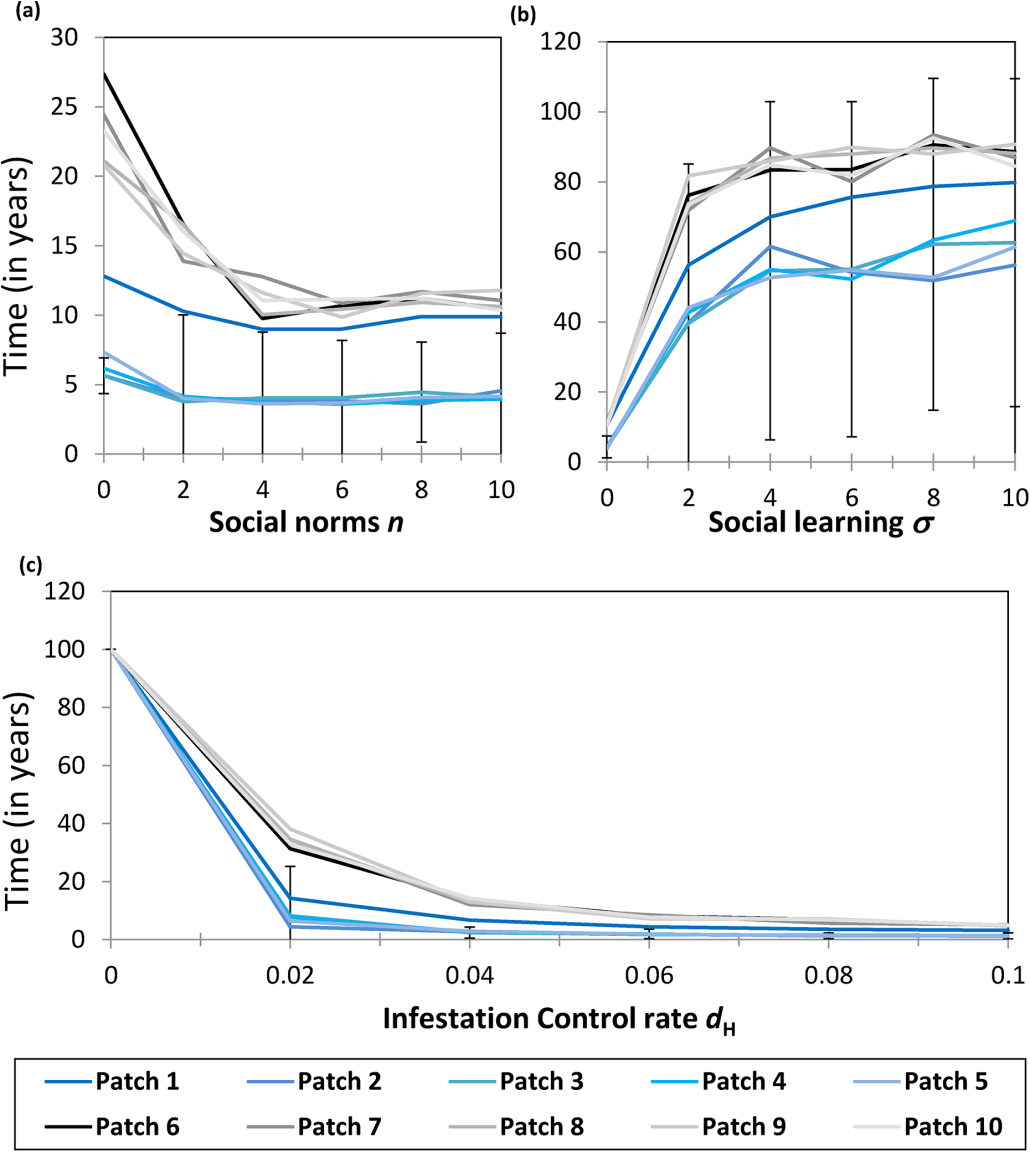
Average of 100 realizations over 100 years’ simulated time. Error bars represent two standard deviations from the mean and have been drawn for only Patch 3 (results are similar for other patches). Patches 1–5 are in Partition 1 while patches 6–10 are in Partition 2.The parameters used to generate the results are given in Table 2. Panels show (a) the impact of social norms *n*; (b) the impact of social learning *σ*; and (c) the impact of changing the volume of visitors in high volume patches *d*_H_ on the average time-to-first-cross–patch-infestation *t*_*cross*_.

In most cases, a change to any of these parameters causes a nonlinear response in the time-to-first-cross–patch-infestation (Figs 4 and 5, data available in the supplementary materials: S2, S3, S4, S5, S6 and S7 Matlab Data Files). In other words, there are many cases where *t*_cross_ does not respond to changes in a parameter’s value, until the parameter value exceeds a threshold, beyond which *t*_cross_ changes dramatically. This has implications for how much effort must be expended to prevent long–range infestation spread, since some changes to control parameters will cause enormous changes in control success, while other changes to control parameters will have little effect on control success. Also, the variability in outcomes (standard deviations) across the 100 model realizations is often significant, meaning that stochasticity could influence whether or not firewood movement restrictions are successful.

For example, decreasing the cost of local firewood (*C*_L_) from the baseline $6.75 per bundle to $4.00 per bundle has a dramatic impact on *t*_cross_, especially for the low volume patches (Fig 4A). As *C*_L_ decreases further, the value of *t*_cross_ reaches a plateau of 100 years. However, this plateau is an artifact caused by imposing 100 years as the simulation duration. Nonetheless, for policy-relevant values of *t*_cross_ (<50 years), these results show that modest decreases in the cost of locally purchased firewood (as provided by park offices, for example) could have significant control benefits.

Outcomes also respond nonlinearly to the fatality probability per unit time, *ε* (Fig 4B). The largest change in *t*_cross_ occurs when the fatality probability increases from 0.4/year to 0.6/year. The baseline value for *ε* is 0.33/year, corresponding to the natural death rate due to infestation. Infection control through tree removal has a similar impact on disease dynamics as tree death. Hence, these results suggest that relatively modest increases in early detection of infested trees, and tree removal efforts, could have a significant impact in preventing cross–patch spread, regardless of their success in preventing localized spread.

Similarly, when the infestation concern parameter *f* is close to zero, small increases *f* result in a rapid increase in *t*_cross_. However, beyond *f* = 0.5, further increases in *f* do not generate the same response (Fig 4C). Increasing *f* helps prevent cross–patch infestation because individuals become local strategists more quickly than when *f* is small. As before, the increase in *t*_cross_ is particularly notable for the low volume partition. Hence, this partition benefit the most from interventions increasing *f*.

Strengthening social norms, *n*, can have a divergent effect on *t*_cross_. When *n* dominates the expressions for the utility *U*_*L*_ and *U*_*T*_, social pressure *per se* plays a more important role than concern for infestation or firewood prices. Hence, if the number of local strategists is not sufficiently numerous to begin with, and if social pressures to conform are sufficiently large, then local strategists will not increase in number, despite infestation or lower firewood costs. This is observed in plots at baseline parameters values where *n* is increased (Fig 5A). However, if initial conditions were such that the number of local strategists was larger than the number of transport strategists, then social norms would pull the population toward having larger numbers of local strategists, and so the outcome would be opposite. Since individuals have less perception of immediate impacts of their actions on others than is the case for second–hand smoking, for instance, we speculate that lower values of *n* are more realistic for firewood movement restrictions; in this case, social norms can influence dynamics, but they do not dominate them.

The social learning probability *σ* has a significant impact on *t*_*cross*_ (Fig 5B). Increasing *σ* facilitates switching between local and transport strategies. If the utilities favor a switch from transport strategists to local strategists (as might occur under conditions of an outbreak where *f·I* is large), then increasing *σ* will increase the number of local strategists more quickly, and thus improves the adoption of firewood movement restrictions. As observed with many other parameters, when *σ* is small, a modest increase in *σ* causes a large increase in *t*_cross_. However, if *σ* is already large, then increasing *σ* still further has little effect on *t*_cross_. Thus, small increases in the social learning rate, as might be brought about by increasing the coverage of forest pest infestations in the media, can have a significant impact on preventing long–distance infestation events. As before, the increases in *t*_cross_ are most significant for the low volume partition, hence remote patches tend to benefit more from increases in *σ*.

Finally, we explore the impact of changing the baseline rate of visits in the high volume partition (*d*_H_) (Fig 5C). A small increase in *d*_H_ causes a significant decrease in *t*_cross_, indicating that efforts to prevent long-distance infestation events can fail if the volume of visitors to parks increases even modestly. The high volume partition suffers the most under the change in *d*_H_, although *t*_cross_ also decreases to a lesser extent for the low volume partition.

## Discussion

In this paper we developed and analyzed a stochastic simulation model of infestation spread due to transportation of infested firewood between 10 patches. The patch geometry was based loosely on the geography of Ontario Parks, with distinct high visitor volume and low visitor volume partitions. Firewood transport was influenced by human decisions based on economic costs, social norms, social learning, and concern about infestations. Our objective was to gain qualitative insights into the nature and implications of nonlinear feedbacks in such systems.

We found that nonlinear responses to changes in control measures and other model parameters were pervasive. For instance, the time-to-first-cross–patch-infestation responded nonlinearity to parameters controlling the cost of locally purchased firewood, the probability per unit time at which infested trees died, the sensitivity of the population to infestation, the probability per unit time of social learning, and the volume of visitors.

In many cases these effects could have implications for policy, especially when the nonlinearities occur near empirically valid parameter values. In particular, modest decreases in the cost of locally purchased firewood *C*_L_ below the current value of approximately $6.75 (the value of which is controlled by parks) caused disproportionate increases in the time-to-first-cross–patch-infestation, meaning that the chances of long–distance spread of infestation were significantly reduced. Similarly, modest increases in the tree mortality probability *ε* over the baseline rate associated with natural death due to infestation also significantly reduced the chances of cross–patch infestation. Hence, timely tree removal efforts may be highly effective in preventing long–distance spread of forest pests, even if they are imperfect in preventing local spread. Increasing the sensitivity to infestation (*f*) and the social learning rate (*σ*) were also effective ways to reduce cross–patch infestation, although the empirical values for these parameters are not as precisely known as is the case for *ε* and *C*_L_. We also found that patches in the low volume partition (distant parks that are visited less often) benefitted more from reduce firewood transportation than patches in the high volume partition. Finally, we observed that the equilibrium levels of infestation were higher in the high volume partition than the low volume partition, despite infestation being endemic in all patches, which implies that subsequent cross–patch infestations have a significant role to play in increasing infestations even when a patch is already infested. In other words, cross–patch infestation is concerning not only because of its seeding effect in starting new infestations, but also because it can worsen existing infestations.

Some of these results echo findings from an earlier 2–patch deterministic model published by Barlow et al. [10], such as the benefits of reducing the cost of local firewood purchased through parks. However, because the current model is a 10–patch stochastic model, we were able to explore effects of stochasticity and park geometry. Barlow et al used a deterministic model, making it difficult to study elimination regimes, and meaning that there was some ambiguity to defining time-to-first-cross-patch infestation (since the infestation is instantaneously transmitted in a deterministic model, unless stochastic effects are artificially captured through imposed thresholds [10]). In contrast, our use of a stochastic model yields an unambiguous definition of how long it takes for the infestation to spread between patches. Moreover, because our model is a 10-patch model instead of a 2-patch model, it was possible to explore issues around park geometry. In particular, we contrasted how time-to-first-cross-patch-infestation responds differently to interventions depending on whether a patch is located in the high volume partition versus the low volume partition. This is not possible in a 2-patch model. Future work could exploit multi-patch geometry more fully by exploring other possible geometries (such as linear or hub-and-spoke).

Policy planning cycles in real populations are much shorter (5–25 years) than the simulation times we used (100s of years). However, long simulation times were part of our experimental design. Our goal in this paper was to obtain a qualitative understanding of the model dynamics, including the inherent tendency of these systems to oscillate on multi-decadal time scales. This provides useful information regarding the fundamental biology of the system, and we would not be able to observe such phenomena in the model if we restricted our simulation time horizon to 25 years. Evaluating long-term dynamics can be useful for informing short-term policy. For instance, policy makers need to be able to properly interpret changes in infestation levels following changes in policy. If the system tends to be naturally stable, then policy makers can be more confident that changes in infestation levels are due changes in their policy. But, if the systems tend to oscillate naturally, then policy makers must be more careful when interpreting changes in infestation levels, since infestation may be declining (or increasing) naturally and not due to changes in policy. Using long simulation time horizons is helpful to establish whether systems tend to naturally oscillate.

As with any model, the current model has limitations. Some of the sociological parameters such as the rate of social learning (*σ*) and the sensitivity to infestation levels (*f*) are not well known; hence we had to use univariate sensitivity analysis. Other features of forest pest outbreaks such as Allee effects and seasonality were not studied. Similarly, we used a relatively simple model of human social behavior, such as classifying all individuals as either local strategists or transport strategists. Also, we did not include a time delay between initial infestation of a tree, and spread of infestation from that tree to un-infested trees upon tree death. Including this delay would probably delay cross-patch infestation by a few years, or perhaps longer depending on how quickly social feedbacks are activated, although we speculate that our conclusions would remain qualitatively unchanged. Many of these simplifying assumptions could be relaxed in future work and could increase the realism of the model, as long as corresponding efforts to obtain empirical estimates of parameter values are also made.

Our goal was to gain a qualitative understanding of coupled human-environment dynamics for forest pests in general, rather than to capture a specific population. However, the model could be modified to capture spread of a particular pest species in Ontario or other jurisdictions. This would require adding more patches to better capture the geometry and connectedness of dozens of regional parks; re-parameterizing the model; and fitting the model to outbreak data from Ontario, in order to test quantitative agreement with data. However, despite the lack of specificity to a particular population, the current model captures qualitative features of forest pest outbreaks, such as more rapid spread to patches that experience a higher volume of visitors, and (unfortunately for the baseline scenario) the insufficient response of the public to prevent cross-patch spread through firewood movement restrictions, in many cases. Future models with greater realism that are designed to capture particular species in particular geographical regions, may assist in the design of control strategies and public messaging campaigns to reduce the destruction caused by invasive forest pests.

## Supporting Information

S1 Matlab Data File (MAT)

S2 Matlab Data File (MAT)

S3 Matlab Data File (MAT)

S4 Matlab Data File (MAT)

S5 Matlab Data File (MAT)

S6 Matlab Data File (MAT)

S7 Matlab Data File (MAT)
